# Permissive and instructive causality: integrating biological and environmental factors in mental health

**DOI:** 10.1017/S003329172610467X

**Published:** 2026-08-07

**Authors:** Igor Branchi

**Affiliations:** 1 Center for Behavioral Sciences and Mental Health, Istituto Superiore Di Sanita, Rome, Italy; 2 National Centre for Drug Research and Evaluation, Istituto Superiore Di Sanita, Rome, Italy

**Keywords:** antidepressants, clinical recovery, complex systems, complexity science, contextual factors, emergence, lifestyle, mental health, mental well-being, plasticity, precision psychiatry, psychedelics, psychotherapy

## Abstract

Psychiatric disorders are complex conditions involving multifactorial, nonlinear causal dynamics, which make effective treatment challenging. To address this complexity, a framework is proposed that distinguishes between types of causality: permissive causality, which pertains to factors affecting the likelihood of an outcome occurring, and instructive causality, which refers to factors shaping the specific features of the outcome. In this framework, when considering an individual as a complex adaptive system, causal processes within the individual are permissive, whereas causal processes outside the individual can be either permissive or instructive in relation to the individual’s global emergent outcomes. Accordingly, when dealing with mental states or complex behaviors, biological substrates – from genes to brain areas – operate through permissive causality, shaping a space of possibilities rather than instructing a specific outcome. By contrast, contextual factors – such as life challenges or subjective appraisal – can also operate through instructive causality, steering behavior and mental states toward specific trajectories. Therapeutic strategies should harness the synergistic interplay between these types of causality, targeting biological substrates to enable transitions in mental states, and leveraging contextual factors to steer these transitions toward mental well-being. Recognizing such interplay holds promise for developing innovative and effective approaches in precision psychiatry, especially for treatments enhancing plasticity – which itself operates through permissive causality – including antidepressants and psychedelics.

## Introduction

Despite significant advancements in neuroscience and psychiatry, effective therapeutic strategies for psychiatric disorders remain elusive for many patients. While current treatments benefit some individuals, a substantial proportion fails to achieve remission, even after multiple interventions (Domschke et al., 2024; Siskind et al., 2022; Trivedi et al., 2006). This significant challenge largely arises from the complexity of causal relationships underlying psychiatric disorders, which emerge from the interplay between biological and environmental factors, with their relative contributions still poorly understood. This complexity is exemplified by the absence of linear cause–effect relationships: the same risk factors or liabilities can lead to different disorders in different individuals, while diverse factors may converge to produce the same condition (Branchi, 2024; Caspi et al., 2014). Furthermore, the principle of dose–response, a cornerstone in many areas of medicine, often proves unpredictable in mental health (Furukawa et al., 2019). These inconsistencies are evident both in the factors that trigger mental illness and in the interventions designed to treat it. Addressing these dynamics requires a paradigm shift toward a conceptual framework that embraces the complexity of psychiatric disorders (Ongur & Paulus, 2024). Such an approach is critical for advancing personalized interventions and improving therapeutic outcomes for individuals living with mental health conditions.

## Psychiatric disorders as complex problems

As for what constitutes a complex problem, Warren Weaver, in his seminal work on complexity (Weaver, 1948), identifies three distinct types of problems: (i) *problems of simplicity*, characterized by linear relationships between pairs of variables, for which a reductionist approach may prove effective for explanation and prediction; (ii) *problems of disorganized complexity*, which involve a large number of elements whose interactions are random or statistical in nature. This randomness allows their overall behavior to be understood and predicted using statistical mechanics and probability theory; and (iii) *problems of organized complexity*, characterized by variables that interact in nonlinear, dynamic, and often unpredictable ways. In this case, the elements of a system are interconnected in nonlinear ways that give rise to emergent properties, such that the functional outcome of the whole system cannot be fully explained by analyzing its individual parts in isolation – a phenomenon that resonates with Aristotle’s notion that ‘the whole is more than the sum of its parts’. Psychiatric disorders often represent problems of emergence, and exploring their causes requires a shift in perspective and methodology away from the strictly reductionist approach.

Applying the perspective of complexity to mental health has profound implications, primarily highlighting that no single biological element, at the genetic, neural, or any level within the individual (Fodor, 1974; Noble, 2012; Putnam, 1967; Sapolsky & Balt, 1996), can be directly and linearly linked to a psychiatric disorder. Instead, mental states emerge from the interplay of multiple levels, encompassing physiological, psychological, and environmental factors (Kendler, 2005). For instance, while genes play a role in conditions like depression or schizophrenia, they do not determine outcomes in isolation. Conversely, environmental factors significantly affect whether and how these disorders manifest. Consequently, understanding psychiatric disorders requires examining the broader networks of interactions among various factors.

However, it is worth noting that adopting this broader network perspective does not imply that every phenomenon within a system, such as an organism, shares the same causal architecture. Rather, within this framework, the relevant system is defined by the focus of inquiry rather than by the highest available level of organization. Thus, although the whole organism can be considered a complex adaptive system, the phenomena investigated within that organism may vary in complexity: a psychiatric condition such as depression may represent a complex emergent condition, whereas a monogenic disorder may be more appropriately treated as a non-complex problem characterized by a relatively direct causal relationship between a biological substrate and the pathological outcome.

The multifactorial nature of psychiatric disorders is well-recognized in contemporary research, and an increasing number of studies emphasize this intricate interplay. As Kenneth Kendler has argued, psychiatry requires an ‘empirically based pluralism’ that accepts the ‘dappled nature’ of causes – ranging from molecular to social – without privileging one level over another (Kendler, 2012). Notably, this multilevel perspective has been further advanced by contemporary network models of psychopathology (Borsboom, 2017). However, managing the multitude of factors causally involved in the emergence of psychiatric disorders and understanding the distinct nature of their relative contributions remains an open issue that has only been partially addressed (Branchi, 2024). Consequently, implementing the theoretical and technical shift required to accommodate this concept within scientific research and clinical settings remains challenging.

## Distinguishing instructive versus permissive causalities

**Figure 1.**
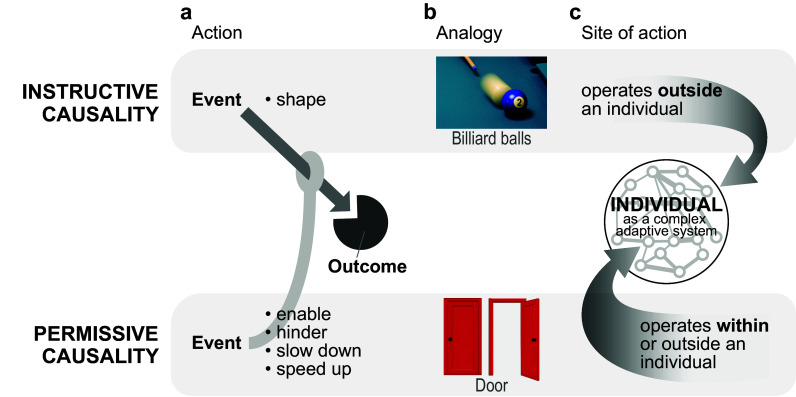
Permissive and instructive causality. *Note*: (a) Instructive causality (top) implies that an action directly determines the quality of its effect, such as through the transfer of energy or information. In contrast, permissive causality (bottom) affects the likelihood of an effect occurring but not its features, for example, by enabling or hindering change, or by speeding up or slowing down transitions. (b) An analogy for instructive causality is the relationship between billiard balls: the motion of one ball changes in response to the specific properties of the ball striking it – such as its speed, angle, or mass. An analogy for permissive causality is that of a door. If it is opened, it makes it possible to pass through it, but this does not imply whether, when, and what will actually pass through. Thus, permissive causality determines the possibility of an outcome to occur but is not tied to the specific nature or quality of that outcome. (c) The two types of causality are predicted to occur differently inside and outside an individual considered as a complex adaptive system interacting with the environment. All elements that lie within the system determine its outcome through permissive causality, whereas factors that are outside the system operate through either instructive or permissive causality. See text for further details. Figure 1. long description.

To tackle the complexity arising from the multitude of factors involved in the onset, progression, and treatment of mental disorders, it is necessary to identify patterns of regularity that clarify the distinct contributions of the factors involved. One such pattern concerns the distinction between different types and domains of causality. Specifically, causal relationships can be classified into two main categories: instructive and permissive (Figure 1). This view emerged independently in different fields such as developmental biology (Calcott, 2017) and psychiatry (Branchi & Giuliani, 2021). *Instructive causality* implies that an action directly determines the specific features of its effect, with causal specificity, such as through the transfer of energy or information (Figure 1a). A telling analogy is the relationship between billiard balls, where the behavior of the target ball changes according to the features of the incoming ball, such as speed, angle, or weight (Figure 1b). In contrast, *permissive causality* affects the likelihood of an effect occurring (Figure 1a). The analogy is that of a door that can be either opened or closed. If the door is open, it makes it possible to pass through it, but this does not determine whether, when, or what will actually pass through (Figure 1b). In other words, permissive causality determines the likelihood of an outcome occurring but is not tied to the specific nature or quality of that outcome. From this perspective, permissive causality provides a conceptual foundation for patterns of effects that, in empirical and statistical models, are often described as moderation (Pearl, 2009; Woodward, 2004).

**Figure 2.**
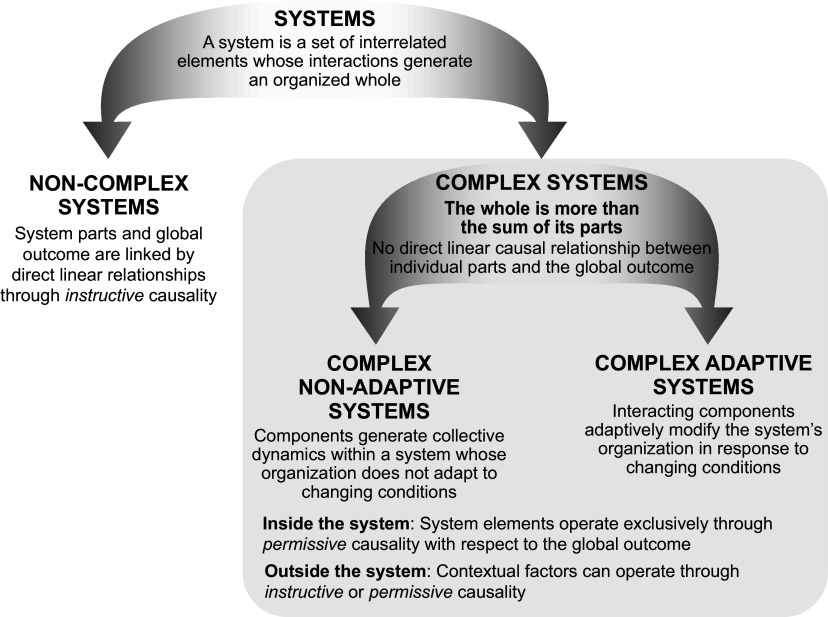
Conceptual taxonomy of systems and their associated causal relationships. *Note*: Systems can be organized according to complexity and adaptivity, with each type characterized by its distinct causal organization. In non-complex systems, system parts and system outcomes are linked by direct linear relationships through instructive causality, analogous to a specific genetic mutation producing a Mendelian disorder. In complex systems, by contrast, whole-level outcomes are not reducible to direct linear causal relationships between individual parts and the global outcome, as illustrated by ecosystems, economies, or the brain, where global states emerge from the interaction of many components. In this case, with respect to the system's global outcome, internal system elements operate exclusively through permissive causality, whereas contextual factors outside the system can operate through either instructive or permissive causality (Branchi, 2022b). The inside/outside distinction becomes operationally clear in complex adaptive systems (Holland, 1995), namely systems that continuously adjust their internal organization in response to contextual conditions, such as organisms. The same logic can be extended to complex non-adaptive systems, provided that the global pattern under investigation is emergent. Even when endogenous processes are self-organizing and driven by internal dynamics that converge on a stable attractor, the self-organized pattern still emerges from interactions among multiple elements, none of which, alone or as a subset, specifies the global outcome in a one-to-one manner. Thus, internal elements operate through permissive causality with respect to the global outcome. See text for further details. Figure 2. long description.

When exploring complex systems, and particularly a complex adaptive system (Holland, 1995) in interaction with its environment, such as an individual, instructive and permissive causalities are predicted to occur differently within and outside the system (Figure 2). All elements that lie within the system are proposed, within this framework, to determine its outcome only through permissive causality, whereas factors that are outside the system can operate through either instructive or permissive causality (Figure 1c). Accordingly, factors internal to the individual may appear as moderators in structural causal models (Pearl, 2009; Woodward, 2004), because they modulate the impact of external events on mental or behavioral outcomes. Within the present framework, this reflects their permissive role in enabling or constraining system dynamics, rather than a lack of causal relevance. More generally, permissive causality can be interpreted as corresponding to factors that shape the dynamical landscape, whereas instructive causality corresponds to perturbations that select specific trajectories within it. This interpretation aligns with recent complex systems models that distinguish between structural landscape properties and state-dependent perturbations acting on the system (Olthof et al., 2023; Scheffer et al., 2024).

This view on the differential occurrence of different types of causality, named the *Context Theory of Constrained Systems* (Branchi, 2024), proposes that, when considering an individual as a complex adaptive system, all factors within the individual – such as genes, molecules, or neural elements – operate exclusively through permissive causality in determining the individual’s outcome, such as a mental state or a behavioral response. By contrast, contextual factors, including environmental challenges and their subjective appraisal, can also exert their effect through instructive causality. This view aligns with the above-mentioned Weaver’s definition of organized complexity, which posits that no component of a complex problem has a linear relationship with the system outcome (Weaver, 1948). Additionally, it resonates with Denis Noble’s influential proposal that no privileged level of causation exists in biology (Noble, 2012). However, in the present framework, this is proposed to pertain specifically to the elements inside the system. Causation within the individual is indeed a distributed process that involves all levels, none of which is inherently more fundamental than the others (Sapolsky & Balt, 1996). Nevertheless, events causing specific outcomes through instructive causality do exist outside the individual (Branchi, 2024), such as hazardous situations that compel people to flee.

### Biological factors operate through permissive causality in determining an individual’s phenotype

Drawing on the definition of complexity grounded in emergence, all elements of a complex system operate through permissive causality with respect to the system’s global outcome, such as the individual’s emergent mental and behavioral phenotypes. This is because, by definition, in an emergent phenomenon, neither any individual element nor any subset of elements specifies the system’s emergent property in a one-to-one manner. When this idea is strictly applied, a bicycle – a system whose capacity for cycling is an emergent property – provides a compelling illustration of the permissive relationship between a system’s elements and its emergent properties: its components – gears, chain, and wheels – constrain and enable the capacity for cycling, yet none of them is linearly responsible for generating that capacity in isolation. At the level of the system’s constituent elements, causality operates by shaping a space of possibilities rather than by instructing a specific outcome. In complex adaptive systems (Figure 2), this inside/outside distinction becomes operationally clear because the system continuously adjusts its internal organization in response to external stimuli and contextual constraints. The same logic also applies to complex non-adaptive systems, provided that the global pattern under investigation is emergent. Even when endogenous self-organization processes converge on stable patterns and give rise to highly constrained global outcomes, the self-organized pattern still arises from interactions among multiple elements, none of which, alone or as a subset, specifies the global outcome in a one-to-one manner. Accordingly, internal manifestations that might appear instructive because of the significant role of self-organization processes, such as hallucinations in psychosis, can be understood as emergent internal states generated by multiple elements operating through permissive causality. Furthermore, hallucinations affect mental states permissively rather than by instructing a specific outcome. This is supported by clinical findings showing that hallucinations are often not directly reduced in frequency or occurrence by treatments like cognitive behavioral therapy, yet their impact is mitigated, demonstrating that such internal states act as moderators rather than deterministic instructions for specific mental states and behavioral phenotypes (Bach & Hayes, 2002; Chadwick & Birchwood, 1994; Garety et al., 2001; Mason, Peters, Williams, & Kumari, 2017).

Crucially, two boundary conditions clarify why internal elements are exclusively permissive regarding complex outcomes. First, regarding the complexity threshold, this framework pertains specifically to complex emergent phenotypes – such as mental states – contrasting with non-complex systems where linear relationships naturally occur. Second, regarding the distinction between local and global causality, we acknowledge that even within a complex adaptive system, specific mechanisms (e.g. a ‘switch’ activating a neural circuit) may operate linearly. However, as these local instructions propagate through the complex network, their linearity is lost through nonlinear interactions, feedback, and context-dependent modulation. By the time they contribute to the global emergent property, their role has transformed from determining a specific effect to modulating the dynamical landscape, and thus effectively becoming permissive. Notably, there are exceptions in which a single biological factor is linearly related to phenotype – such as in Mendelian genetic disorders – but these are not problems grounded in emergence and can be successfully addressed with a reductionist, linear approach.

### Evolutionary processes embed instructive influences within the system as endogenous constraints

The evolutionary process is critical in embedding instructive influences from past environments within the individual, stabilizing the underlying architectures that support behavioral and mental dispositions through endogenous patterns of self-organization. When specific features prove effective over generations, these architectures can become genetically embedded, yielding species-specific, partially innate propensities (Tinbergen, 1951). However, these responses remain fundamentally dependent on external factors primarily for three reasons: first, they are typically triggered by external stimuli. Second, they yield reliable outcomes only in a stable environment where the conditions for their execution remain consistent with the outcomes they evolved to produce. Third, should the environment prove unstable, the embedded patterns of activation cease to specify a fixed response and instead become effectively permissive, engaging with other factors in the production of a response that adapts to the changing environment. When this shift fails to occur, a misalignment between endogenously instructed behavioral responses or mental states and the environment arises, resulting in dysfunctional outcomes that may predispose to mental disorders (Nesse, 2023).

It is noteworthy that the distinction between instructive and permissive causality – and their respective domains of action outside and within the individual – can be interpreted as resonating with Darwin’s theory of natural selection (Darwin, 1859). In the Darwinian theory, the evolutionary process relies on three core phases: variation, selection, and retention. Environmental conditions present challenges that favor some responses over others, representing the phase of selection and corresponding to the concept of instructive causality operating from outside the individual. Conversely, the repertoire of possible responses is determined by the individual’s internal capacities, representing the phase of variation and paralleling permissive causality operating from inside. Finally, the cycle is completed by retention, the mechanism by which selected variants are stabilized and preserved as endogenous patterns of self-organization.

### Epigenetic embedding of instructive influences within the lifespan

While evolution operates over generations, the interplay between instructive and permissive causality is mirrored within the lifespan by epigenetic processes, which can be understood as the biological embedding of environmental experiences (Aristizabal et al., 2020). In this framework, environmental exposures can operate in an instructive manner by providing specific signals – such as early-life stress or nurturing – that shape chromatin-based modifications (e.g. DNA methylation and histone modifications) and thereby influence gene regulation (Meaney & Szyf, 2005) and gene × environment interplay (Klengel & Binder, 2015), with downstream consequences for mental and behavioral outcomes. Importantly, once established, these epigenetic modifications can persist over time and become part of the individual’s internal biological substrate. At this stage, their causal role is best described as permissive with respect to emergent clinical phenotypes: epigenetic marks typically do not linearly determine a specific psychopathological outcome (Nestler, 2014), but rather bias gene-expression programs and constrain the range of possible regulatory states (Klengel & Binder, 2015). This can alter an individual’s susceptibility, changing the likelihood that future environmental challenges will increase the probability of a psychopathological outcome without uniquely specifying its form. Thus, epigenetics provides a concrete example of how contextual information can be biologically transduced and stabilized, bridging context and biology by transforming externally driven signals into internally instantiated constraints that subsequently operate permissively with respect to emergent clinical phenotypes, in parallel with the way evolutionary processes transform past environmental selection pressures into internal constraints across generations.

## Distinct therapeutic actions of instructive and permissive causality

**Figure 3.**
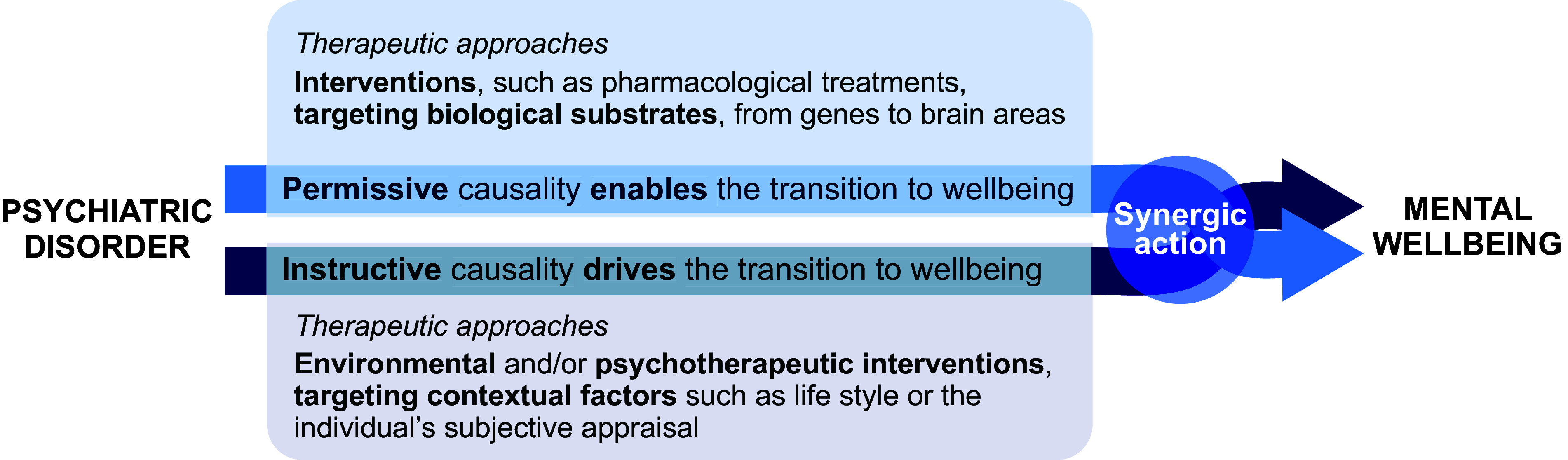
The synergistic action of permissive and instructive causality in the transition from psychiatric disorders to mental well-being. *Note*: The complexity of psychiatric disorders can be addressed by distinguishing between permissive and instructive causality. Permissive causality pertains to causes affecting the likelihood of an outcome, while instructive causality refers to causes that drive the specific features of the outcome. Biological substrates – ranging from genes to brain areas – operate through permissive causality, as they enable transitions in mental states. By contrast, contextual factors, such as lifestyle or the individual’s subjective appraisal, also operate through instructive causality, driving the transition toward mental well-being. Consequently, therapeutic strategies can be most effective when they integrate both types of causal action, combining interventions that enable transitions with interventions that steer them toward the clinical goal. Figure 3. long description.

When applying instructive and permissive causalities to psychiatry, it is essential to consider that both biological and contextual factors are equally critical in determining mental states and behaviors. However, they play antithetical roles: on the one hand, biological substrates, from genes to brain areas, enable mental or behavioral outcomes. On the other hand, contextual factors – such as life challenges – trigger the outcome to be produced (Branchi, 2024) (Figure 3). Using an analogy: I can talk because I have Broca’s area in my brain (i.e. permissive causality, occurring within the individual), but I speak because someone is listening (i.e. instructive causality and occurring outside). This view has been exemplified through the *kitchen metaphor* of the brain (Branchi, 2024), where the ingredients (i.e. biological substrates) provide the means to prepare a recipe, but the circumstances (i.e. contextual factors) dictate which recipe is ultimately made. The following paragraphs will describe how the distinction between permissive and instructive causality can inform the development of innovative and effective therapeutic strategies capable of better addressing the complexity of the brain and the mind. It is noteworthy that this theoretical framework applies specifically to complex psychiatric disorders. By contrast, non-complex disorders can often be effectively addressed using a reductionist approach, focusing on linear cause–effect relationships (Branchi, 2024).

### Targeting biological substrates

Biological substrates, including genes, molecules, and neural elements, are structurally and functionally essential because they serve as building blocks for producing mental states and behavior. However, they do not dictate which outcomes occur (Branchi, 2022b), but rather, through permissive causality, they determine which outcomes can be afforded. Indeed, since the brain is a complex adaptive system, deleting or adding any element of the brain does not typically have a specific and linear consequence on mental states, but it will modify the range of possible mental outcomes. If any biological substrate is missing or impaired, due to genetic mutations, lesions, or other events, a specific mental or behavioral outcome may no longer be affordable because the necessary elements to produce it are no longer available. Nevertheless, an alternative response still must be produced because of the need to cope with the contextual challenges. Such an alternative response will be selected on the basis of the biological substrates still available and may not be directly related to the missing substrate. Thus, biological elements should be considered not as drivers of a specific phenotype but as constraining factors in brain functioning and, consequently, in the production of mental and behavioral outcomes (Branchi, 2024).

When translating this view to clinical settings, it follows that treatments targeting biological substrates, such as pharmacological approaches, are unlikely to produce a specific outcome, although they do modify the likelihood of its occurrence, via permissive causality (Branchi, 2022b; Edelman & Gally, 2001; Prinz, Bucher, & Marder, 2004). Accordingly, a growing body of research demonstrates that genetic risk factors and liabilities (Bassett, Scherer, & Brzustowicz, 2010; Kendler, Prescott, Myers, & Neale, 2003; Zhu, Need, Petrovski, & Goldstein, 2014) and drugs targeting specific substrates (Ford & Young, 2022; Schneider et al., 2025; Viglione et al., 2019) do not produce univocal effects but instead create a range of potential outcomes. It is noteworthy that this view does not apply to disorders that can be considered non-complex systems, which exhibit instructive relationships between specific substrates and outcomes. Examples of non-complex disorders are Mendelian genetic diseases. Moreover, while most psychiatric disorders are complex, some psychiatric syndromes are secondary to relatively circumscribed biological determinants, such as Pellagra (driven by niacin deficiency) or Phenylketonuria (driven by a single gene mutation), where a primary cause is predictably linked to the pathological outcome.

### Targeting context

In complex problems, such as psychiatric disorders, instructive causality is expected to arise only in the interaction with context (Branchi, 2024). Therefore, contextual factors should be considered not as passive players affecting the outcome of a ‘diseased’ brain but as active determinants capable of controlling and orchestrating brain functioning. Thus, therapeutic strategies based on the modification of the objective features of the environment, or targeting the individual’s subjective appraisal, represent powerful tools to reorganize the central nervous system, promoting mental well-being (Branchi, 2022b). Environmental interventions, such as green care or social farming (Borgi et al., 2019; Vera, 2020), and psychotherapy, which modifies how the individual copes with context (Cuijpers et al., 2020; Grunder et al., 2024), are increasingly proven effective in the treatment of psychiatric disorders. Accordingly, differences in subjective appraisal have been demonstrated to determine the outcome of psychiatric treatments, including drug administration and neuromodulation (Dutcher & Krystal, 2024; Fassi et al., 2024; Godlewska et al., 2016; Shiroma, Thuras, Johns, & Lim, 2014; Wardenaar, Conradi, Bos, & de Jonge, 2014). Furthermore, psychotherapy has been demonstrated to impact biological substrates not only in brain disorders (Dichter, Felder, & Smoski, 2010; Fu et al., 2008; Mason et al., 2017) but also in diseases affecting the entire body, such as cancer (Antoni et al., 2006; Borgi, Collacchi, Ortona, & Cirulli, 2020; Chida, Hamer, Wardle, & Steptoe, 2008; Spiegel, 2002). One of the most compelling examples of the critical role played by contextual factors in driving the organization of biological processes is the placebo effect (Benedetti, 2008), where the patient’s expectation of clinical benefit is essential (Andrews, 2001; Bargmann, 2012; Colloca & Benedetti, 2005). As such, the placebo effect is a biological phenomenon that emerges from the individual’s psychosocial and emotional context (Benedetti, 2008). Pain perception is one of the key examples of the placebo response (Price et al., 1999; Swerts, Benedetti, & Peres, 2022). Additionally, in psychiatry, studies have shown that the response to antidepressant treatments is significantly influenced by the placebo response (Cipriani et al., 2009; Kirsch et al., 2008; Stone et al., 2022; Walsh, Seidman, Sysko, & Gould, 2002).

Despite its importance, the individual’s context – that is, the subjective experience of the environment according to personal history and features – is often overlooked in clinical research and mental health practice. This oversight may significantly undermine the reliability, explanatory power, and impact of studies aimed at understanding psychopathology and developing new treatments. Though there are notable exceptions (Trivedi et al., 2006), clinical trials in psychiatry rarely consider detailed contextual information, potentially failing to identify events and processes of clinical significance that could benefit patients (Schumann et al., 2014).

## Combining instructive and permissive causality in treatment design

Given their complex nature, psychiatric disorders can be effectively addressed by considering the different actions of instructive and permissive causality (Branchi, 2024). Some individuals may struggle to achieve well-being due to a lack of necessary elements for recovery, such as specific levels of neurotransmitters or other neural elements. In such cases, pharmacological treatments can restore a condition that – through permissive causality – enables recovery. However, reinstating this condition is necessary but may not be sufficient. Indeed, even when possessing the requisite neural elements, individuals may fail to recover because they are not experiencing contextual factors that can promote well-being, which operate through instructive causality (Branchi, 2025). Thus, addressing only one type of causality may not be sufficient to determine a beneficial outcome. Effective therapeutic strategies should combine contextual interventions, which provide instructive signals that drive the patient’s mental state toward well-being, with targeting biological substrates that enable the brain changes driven by the contextual intervention. It is increasingly shown that their combination increases the efficacy of therapeutic approaches for mental illness. For instance, this has been shown for selective serotonin reuptake inhibitors in depression (Chiarotti, Viglione, Giuliani, & Branchi, 2017; Cuijpers et al., 2020), as well as for oxytocin administration in autism spectrum disorder (Ford & Young, 2022).

## Plasticity operates through permissive causality: implications for clinical practice

Permissive and instructive causality offer a valuable theoretical framework for understanding the therapeutic action of psychiatric treatments that promote well-being through plasticity enhancement. Plasticity is defined as the capacity to modify brain functioning, behaviors, and mental states (Branchi, 2011, 2026). It is increasingly acknowledged as a crucial process in mental health because it underlies the reorganization of brain functioning and mental processes during the transition from psychopathology to well-being (Branchi, 2022a). The definition of plasticity implies that it is neither inherently beneficial nor detrimental because it operates through permissive causality (Figure 1), affecting the likelihood of a transition without setting its direction. The direction is determined by moderators, including contextual factors such as living conditions or the subjective appraisal of quality of life, which operate through instructive causality (Branchi & Giuliani, 2021). It is noteworthy that, while plasticity operates permissively at the level of mental states and symptom trajectories, the biological processes that implement plastic change can be highly specific, targeting selected circuits, synapses, or gene-regulatory programs in response to experience. A growing body of evidence increasingly suggests that treatments that enhance plasticity produce context-dependent effects, amplifying the influence of contextual factors in shaping mental health and behavioral outcomes. Consequently, high plasticity has a relevant therapeutic impact when combined with supportive living conditions or psychotherapeutic approaches (Branchi, 2011; Branchi, 2024) and is consistent with the ‘differential susceptibility’ framework, which proposes that higher plasticity increases sensitivity to environmental influences ‘for better and for worse’ (Belsky et al., 2009; Ellis & Boyce, 2008). Indeed, the efficacy of treatments such as selective serotonin reuptake inhibitors (SSRIs), reportedly enhancing plasticity, is partially dependent on their pairing with favorable environmental conditions (Chiarotti et al., 2017; Klobl et al., 2022; Viglione et al., 2019). Accordingly, the combination of antidepressants with psychotherapy is more effective than the drugs alone (Carhart-Harris et al., 2018; Cuijpers et al., 2021; Grunder et al., 2024).

### Plasticity-enhancing treatments: context is essential for therapeutic efficacy

The theoretical framework proposed here holds critical implications in clinical practice, particularly for novel potential treatments for depression such as psychedelics, 3,4-methylenedioxymethamphetamine, and ketamine. Since their therapeutic action relies, at least in part, on the ability to enhance plasticity, their efficacy emerges from the combination with contextual factors promoting well-being. Indeed, they have been shown to produce effects that are affected by the psychological context, and thus by the placebo effect (Bottemanne et al., 2022; Dutcher & Krystal, 2024; Mitchell et al., 2023; R. B. Price et al., 2022; Seybert et al., 2025). Therefore, these treatments may be understood as adjuncts to psychotherapy or environmental interventions. Attempts to isolate the pharmacological effect from the contextual one could fail to capture the potential of these treatments in real-world settings, and prescribing them without controlling for the context in which they are administered may undermine their therapeutic efficacy. Despite valid concerns from regulatory agencies, which view psychotherapy-assisted drug treatments as overly complex and challenging to regulate (Kupferschmidt, 2024; Reardon, 2024), the theoretical framework proposed here highlights that the beneficial outcomes of plasticity-enhancing treatments can be achieved only by integrating contextual factors into the therapeutic approach. This is because exploiting both instructive and permissive causality is essential for achieving well-being, as relying on only one type of causality may not be sufficient. In other words, as plasticity is not inherently beneficial by definition, treatments aimed at enhancing it do not, *per se* , have a predictable therapeutic effect.

## Conclusions

Traditional models based on linear and one-to-one relationships between specific biological substrates and mental outcomes often fall short of capturing the causal structure of the interplay between the brain, the individual’s context, and mental health. To overcome this limitation, contemporary research integrates theoretical and empirical perspectives to address causality (Kendler & Campbell, 2009; Ross & Woodward, 2023; Woodward, 2004). Such developments align with recent appeals in neuroscience for a richer causal lexicon, moving beyond a single definition to capture the diverse ways events influence one another (Barack et al., 2022). This perspective acknowledges that psychiatric disorders emerge from intricate interactions among diverse factors across multiple levels, including genetic, neural, psychological, and environmental influences (Bolton, 2023; Branchi, 2024; Kendler, 2012; Ross, 2019). Accordingly, causality has been redefined as emerging from dynamic interactions among interconnected elements, marking a shift from latent-variable to network-based models that more precisely capture the nature of mental disorders (Borsboom, 2017).

The recognition of the multifactorial origin of complex mental conditions is extended here by proposing that biological substrates and contextual factors not only interact in the onset and treatment of psychopathology but also play distinct roles in these processes. Distinguishing between instructive and permissive causality, and clarifying their different domains of operation, provides a way to specify these distinct roles and can therefore help address the complexity of multifactorial causation and offer deeper insights into the processes underlying mental health. In other words, therapeutic action in mental health must leverage both biological and contextual factors, not only because they are both relevant for well-being but also because they play different and synergistic roles in the recovery process.

Overall, the theoretical framework proposed here moves beyond acknowledging that both biological and environmental factors participate in a nonlinear and interactive regulation of the onset, course, and treatment of mental illness, as suggested by the biopsychosocial and other models (Bolton, 2023; Engel, 1980). It also specifies that these factors determine disease trajectories through distinct causal mechanisms, thereby paving the way for innovative therapeutic strategies and offering the potential for more effective approaches in precision psychiatry.

## Figure 1. Long description

The diagram is divided into three labeled columns a, b, and c, and two horizontal sections for instructive causality (top) and permissive causality (bottom). In column a, labeled Action, instructive causality shows an arrow from Event to Outcome with the label shape, while permissive causality shows an arrow from Event to Outcome with the labels enable, hinder, slow down, speed up. In column b, labeled Analogy, instructive causality is illustrated by a photo of billiard balls with the label Billiard balls, and permissive causality by two doors, one closed and one open, with the label Door. In column c, labeled Site of action, instructive causality is linked by a curved arrow to the phrase operates outside an individual, while permissive causality is linked by a curved arrow to a circle labeled INDIVIDUAL as a complex adaptive system, with the phrase operates within or outside an individual. The arrows and layout emphasize the differences in how each causality type acts, their analogies, and their relationship to system boundaries.

Navigate back to Figure 1..

## Figure 2. Long description

At the top, a rounded rectangle labeled SYSTEMS defines a system as a set of interrelated elements whose interactions generate an organized whole. Three arrows point downward. The left arrow leads to NON-COMPLEX SYSTEMS, described as having system parts and global outcomes linked by direct linear relationships through instructive causality. The center arrow leads to COMPLEX SYSTEMS, defined as systems where the whole is more than the sum of its parts, with no direct linear causal relationship between individual parts and the global outcome. From COMPLEX SYSTEMS, a downward arrow leads to COMPLEX NON-ADAPTIVE SYSTEMS, which are described as having endogenous self-organizing processes that operate through instructive causality in determining the system global outcome. The right arrow from SYSTEMS leads to COMPLEX ADAPTIVE SYSTEMS, which are divided into two sections: Inside the system, system elements operate exclusively through permissive causality with respect to the global outcome; outside the system, contextual factors can operate through instructive or permissive causality.

Navigate back to Figure 2..

## Figure 3. Long description

At the left, Psychiatric Disorder anchors two horizontal bands. The upper band describes permissive causality, labeled as enabling the transition to wellbeing, with therapeutic approaches targeting biological substrates such as pharmacological treatments from genes to brain areas. The lower band describes instructive causality, labeled as driving the transition to wellbeing, with therapeutic approaches targeting contextual factors such as environmental or psychotherapeutic interventions, lifestyle, or subjective appraisal. Both bands converge at a central blue circle labeled Synergic Action, which points rightward to Mental Wellbeing.

Navigate back to Figure 3..
